# Secondary Metabolites of Plants as Modulators of Endothelium Functions

**DOI:** 10.3390/ijms22052533

**Published:** 2021-03-03

**Authors:** Anna Bartáková, Marie Nováková

**Affiliations:** 1 Department of Physiology, Faculty of Medicine, Masaryk University, 625 00 Brno, Czech Republic; anna.bartakova@med.muni.cz; 2 International Clinical Research Center, St. Anne’s University Hospital Brno, 656 91 Brno, Czech Republic

**Keywords:** endothelium, vasoactive substances, vasodilation, vasoconstriction, nitric oxide

## Abstract

According to the World Health Organization, cardiovascular diseases are the main cause of death worldwide. They may be caused by various factors or combinations of factors. Frequently, endothelial dysfunction is involved in either development of the disorder or results from it. On the other hand, the endothelium may be disordered for other reasons, e.g., due to infection, such as COVID-19. The understanding of the role and significance of the endothelium in the body has changed significantly over time—from a simple physical barrier to a complex system encompassing local and systemic regulation of numerous processes in the body. Endothelium disorders may arise from impairment of one or more signaling pathways affecting dilator or constrictor activity, including nitric oxide–cyclic guanosine monophosphate activation, prostacyclin–cyclic adenosine monophosphate activation, phosphodiesterase inhibition, and potassium channel activation or intracellular calcium level inhibition. In this review, plants are summarized as sources of biologically active substances affecting the endothelium. This paper compares individual substances and mechanisms that are known to affect the endothelium, and which subsequently may cause the development of cardiovascular disorders.

## 1. Introduction

According to the World Health Organization (WHO), almost 18 million people died worldwide in 2017 due to cardiovascular disorders. Numerous experimental and clinical studies are, therefore, focused on the cardiovascular system under both physiological and pathological conditions.

The cardiovascular system consists of the heart and vessels of various types. Three layers form a typical vessel: the tunica intima, tunica media, and tunica adventitia. The thickness ratio of a vessel wall depends on the functional requirements of that particular part of circulation system. Nevertheless, endothelial cells are a standard part of the tunica intima in any vessel.

## 2. The Endothelium: From a Simple Barrier to a Specialized Organ

### 2.1. Morphology of the Endothelium

A single layer of flat endothelial cells covers the inner surface of a vessel, which is in direct contact with the blood. Thus, this inner lining provides an anticoagulant barrier between the vessel wall and blood. All endothelial cells form a large organ consisting of approximately 1–6 × 10^13^ of cells, a mass of almost one kilogram [1].

The endothelium originates from the splanchnopleuric mesoderm [1]. Vascular endothelial growth factor (VEGF) and its high-affinity flk-1 and flt-1 receptor tyrosine kinases represent a paracrine signaling system that is critical for endothelial cell differentiation and vascular system development [2,3]. It has been proven that VEGF is the only specific mitogen for endothelial cells. It stimulates their growth, inhibits apoptosis, increases vascular permeability in various tissues, and promotes vasculogenesis and angiogenesis. Angiogenesis plays a protective role in coronary artery disease and myocardial infarction [4].

**Figure 1:**
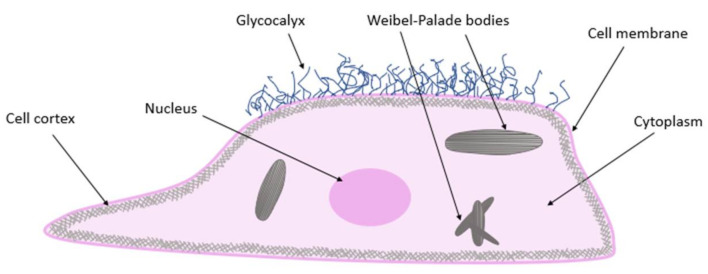
Endothelial cell structure.

Endothelial cells consist of four basic compartments: the glycocalyx, cell cortex, cytoplasm, and nucleus (Figure 1). The structure and mechanical properties of these compartments directly affect physiological processes [1]. The endothelial glycocalyx is a thick, carbohydrate-rich layer that surrounds the endothelial lumen surface; it is composed of proteoglycans and glycoproteins. On the inner side of a cell membrane, the cell cortex is found, containing actin organized in a dynamic net. Actin fibers represent a support network for the plasma membrane and membrane proteins. The cell is also penetrated by actin microtubules and intermediate filaments. All components of the cell cytoskeleton are associated with the nucleus. Mechanical stimuli perceived by actin fibers, microtubules, or intermediate filaments are integrated in the nucleus [5]. Endothelial cells contain so-called Weibel–Palade bodies, measuring 0.1 µm wide and 0.3 µm long. These membrane-bound structures are a kind of storage organelle for von Willebrand’s factor (vWf) (Figure 1) [1].

### 2.2. Physiological Roles of Endothelium

For a long time, the role of the simple barrier was attributed to the endothelium. Since then, its concept has changed significantly and new functions of endothelial cells have been reported. It is now considered a specialized organ with numerous physiological functions [1].

First of all, the barrier function of the endothelium is viewed in a less static way than in the original concept, where the endothelium was believed to simply separate blood from the surrounding tissues. Nowadays, it is considered a dynamic barrier, the integrity of which is essential for maintaining physiological blood flow. On the other hand, endothelial cells communicate among themselves on one side and with circulating blood elements on the other side; the latter involves thrombocytes and leukocytes. Communication with other cells, even distant ones, via various paracrine and endocrine substances has also been described. All of these cells, cooperatively with the blood flow, affect the behavior of the endothelium [6].

Based on the above, it can be presumed that both endothelial cell injury and its dysfunction may lead to a number of pathological situations. Endothelial dysfunction results in various seemingly unrelated pathological processes, such as loss of semipermeable membrane function, hyperlipoproteinemia (often accompanied by atherogenesis), diabetes mellitus, vascular spasms, and arterial hypertension. Together with certain risk factors (e.g., smoking), these processes progress to uniform vascular changes. Subsequent organ hypoperfusion leads to failure in the target structure, for example heart failure [1].

**Figure 2:**
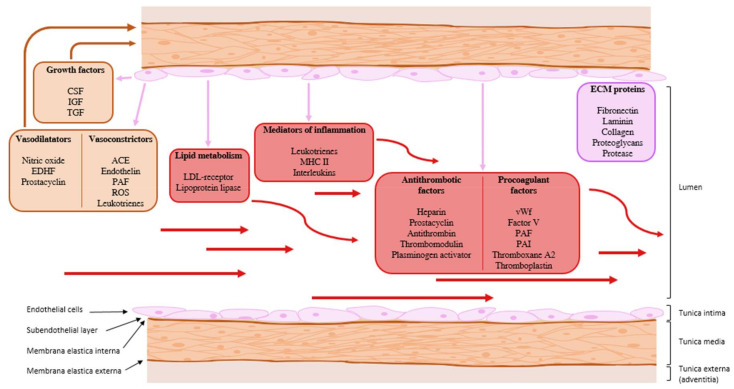
The basic humoral and metabolic functions of the endothelium. ACE: angiotensin converting enzyme; CSF: colony-stimulating factor; ECM: extracellular matrix; EDH: endothelium-derived hyperpolarization; IGF: insulin-like growth factor; LDL receptor: low-density lipoprotein receptor; MHC II: major histocompatibility complex type 2; PAF: platelet-activating factor; PAI: plasminogen activator inhibitor; ROS: reactive oxygen species; TGF: transforming growth factor; vWf: von Willebrand’s factor. Purple arrow: paracrine communication, red arrow: endocrine communication.

The basic humoral and metabolic functions of the endothelium are summarized in Figure 2. Various types of autocrine, paracrine, and endocrine communication systems are presented.

#### 2.2.1. Vascular Tone Regulation

**Figure 3:**
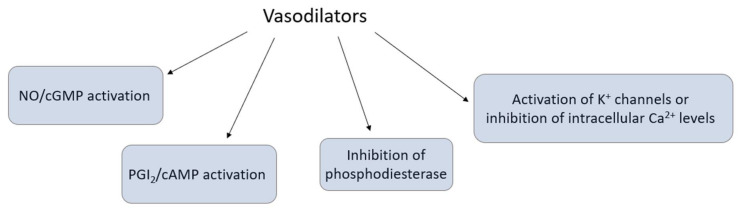
Endothelial regulation of vascular tone via several pathways.

The endothelium is a site of production or modification of numerous vasodilatory and vasoconstrictory substances, which regulate the vascular tone via several pathways, namely nitric oxide–cyclic guanosine monophosphate (NO–cGMP) activation, prostacyclin–cyclic adenosine monophosphate (PGI_2_–cAMP) activation, inhibition of phosphodiesterase (PDE), and activation of K^+^ channels or inhibition of intracellular Ca^2+^ levels (Figure 3).

The endothelial cell reacts to physical and chemical stimuli from the circulation. Physical (hemodynamic) factors increase the sensory tension of endothelial cells, which depends on the blood flow velocity in the vessels. Chemical stimuli are represented by vasoactive substances (e.g., adenosine monophosphate, bradykinin, histamine), neurotransmitters (e.g., acetylcholine), hormones (e.g., antidiuretic hormone, angiotensin), coagulation factors, and substances produced by platelets (e.g., thrombin) [1].

In cases of locally increased blood flow, the local regulatory system is activated, which results in endothelium-mediated vasodilation. Nitric oxide (NO), prostacyclin (PGI_2_), or endothelium-derived hyperpolarization (EDH) is secreted from the endothelium due to the increased shear stress. This may be a form of endothelium protection, resulting from increased blood flow. In the case of a turbulent flow, the risk of damage to the endothelium and consequent thrombus formation increases. NO mainly regulates the tonus of relatively large conduit vessels. On the contrary, EDH mediates vasodilation, especially in small resistance vessels in the microcirculation. Prostacyclins play a small but constant role, independent of vessel size. Furthermore, metabolic regulation can occur when substances (e.g., O_2_) that are necessary to ensure metabolism or emerging catabolites (CO_2_, lactic acid, adenosine, and others) act on vascular smooth muscle and affect its tone, either directly or more often through endothelial receptors [7,8,9].

##### Angiotensin-Converting Enzyme

**Figure 4:**
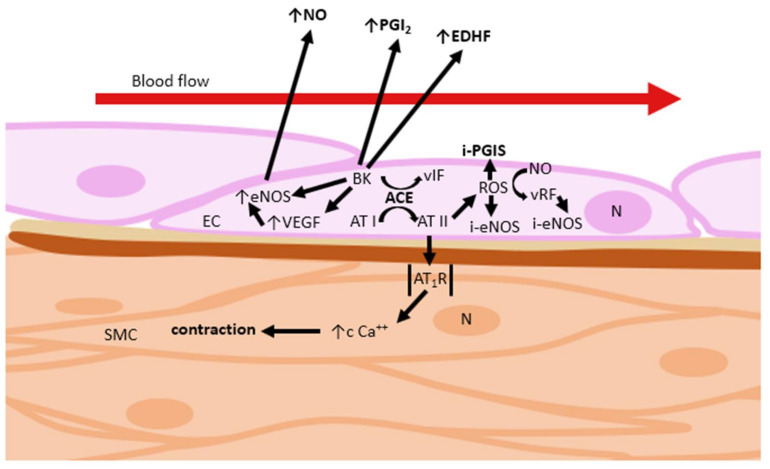
Regulation of vascular tone via ACE pathway. AT I: angiotensin I; AT II: angiotensin II; ACE: angiotensin-converting enzyme; AT_1_R: angiotensin type-1 receptor; BK: bradykinin; EC: endothelial cell; eNOS: endothelial NO synthase; i-eNOS: endothelial NO synthase inhibition; i-PGIS: prostacyclin synthase inhibition; NO: nitric oxide; N: nucleus; ROS: reactive oxygen species; SMC: smooth muscle cell; vIF: various inactive fragments; vRF: various reactive fragments.

A detailed view of the intracellular mediation of the effects of vasoactive substances brings about a thought-provoking idea: a key player in this game is angiotensin-converting enzyme (ACE), also known as kininase II. It is produced by the vascular endothelium and plays a central role in the renin–angiotensin–aldosterone system (RAAS). ACE converts angiotensin I (AT I) to octapeptide angiotensin II (AT II), which is a very potent vasoconstrictor (Figure 4) [10]. AT II increases the production of reactive oxygen species (ROS) via increasing NADPH oxidase activity. Increased levels of endothelial ROS lead to rapid inactivation or degradation of NO, and at the same time to endothelial nitric oxide synthase (eNOS) and prostacyclin synthase (PGIS) inhibition [10,11,12,13,14]. It is important to mention that NADPH oxidase activation is one of the pathways involved in production of endothelium-derived H_2_O_2_ (E-D H_2_O_2_) hyperpolarizing factor, a substance with high vasodilating potency [7].

AT II itself increases blood pressure, not only through vasoconstriction, but also through stimulation of the sympathetic system via the synthesis of aldosterone. AT II also acts as an inducer of growth, cell migration, and cell mitosis in vascular smooth muscle. It also increases the synthesis of type I and III collagen in fibroblasts, resulting in thickening of the blood vessel wall and myocardium and fibrosis. These effects are mediated by receptor type I for angiotensin II (AT_1_R) and can be blocked by AT_1_R blockers known as the “sartan” family [15,16]. Receptor type II for AT II mediates the opposite effect, e.g., inhibition of cell proliferation in coronary endothelial cells [17]. AT II may trigger endothelial cell apoptosis, mediated either by generation of ROS or by inhibiting the function of the antiapoptotic protein B-cell lymphoma 2 [11]. The regulation of its effect is an essential part of the clinical practice of treating hypertension [10].

Moreover, ACE degrades kinins. Bradykinin stimulates NO and PGI_2_ release [10,11,12,14] and increases vascular permeability [18]. The effect of bradykinin on NO release is mediated by B_2_ receptor [10,11,12,14]. Angiotensin-converting enzyme inhibitors (iACEs) potentiate the actions of bradykinin by reducing its degradation [11], which leads to higher bradykinin levels. On the contrary, blocking the effect of AT II through AT_1_R does not affect the level of bradykinin [19].

At this point, we would like to emphasize that iACEs affect the delicate physiological balance between NO and EDH [7].

##### Nitric Oxide–Cyclic Guanosine Monophosphate Activation Pathway

Endothelium-derived relaxing factor (NO) is produced from the amino acid arginine, which is transferred into the amino acid citrulline. This reaction is catalyzed by the enzyme nitric oxide synthase (NOS).

**Figure 5:**
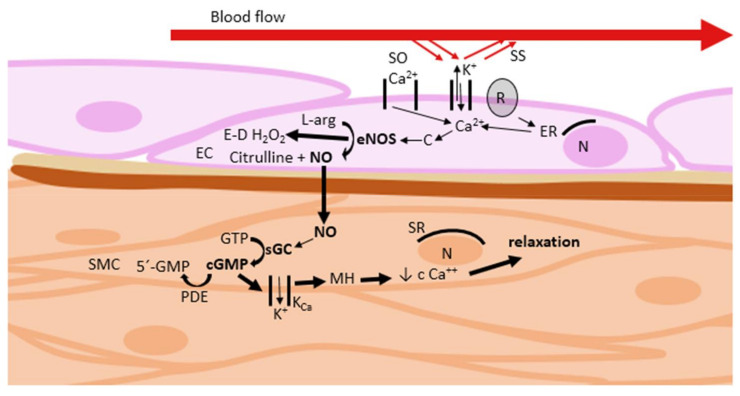
Regulation of vascular tone via nitric oxide–cyclic guanosine monophosphate pathway. C: calmodulin; cGMP: cyclic guanosine monophosphate; EC: endothelial cell; E-D H_2_O_2_: endothelium-derived H_2_O_2_; eNOS: endothelial NO synthase; ER: endoplasmic reticulum; GTP: guanosine triphosphate; K_Ca_: Ca^2+^-activated K^+^ channels; L-arg: L-arginine; MH: membrane hyperpolarization; N: nucleus; NO: nitric oxide; PDE: phosphodiesterase; R: receptor; sGC: soluble receptor with guanylate cyclase activity; SMC: smooth muscle cell; SO Ca^2+^: store-operated Ca^2+^ channels; SR: sarcoplasmic reticulum; SS: shear stress.

Nitric oxide is one of the three gasotransmitters, along with carbon monoxide (CO) and hydrogen sulphide (H_2_S), which are critical for cardiovascular homeostasis [20]. NO acts as a mediator, having a local vasodilatory effect on vascular smooth muscle. NOS exists in three isoforms: endothelial (eNOS), neural (nNOS), and inducible (iNOS). Vascular tone regulation is primarily dependent on NO produced in the reaction catalyzed by eNOS [21,22]. Its production is regulated either at the level of its activity (increased by agonists such as CO, bradykinin, acetylcholine, substance P, thrombin, insulin, and shear stress) or gene expression [6,21,22,23,24,25]. NO stimulates the soluble receptor with guanylate cyclase activity (sGC) in a neighboring cell. This leads to an increase in the cyclic guanosine monophosphate (cGMP) concentration, and consequently to vasodilation (Figure 5). Another possible way to affect the NO–cGMP pathway is to modulate the activity or gene expression of sGC. Some substances activate the sGC [21,22].

Inhibitors of both eNOS and sGC are used in studies focusing on the NO–cGMP pathway. In the case of eNOS, NG-nitro-L-arginine methyl esters or NG-monomethyl-L-arginine are most often used; in the case of sGC, methylene blue or 1*H*-[1,2,4]oxadiazole[4,3-a]quinoxalin-1-one can be employed [21,22]. Another possible approach is the use of NO scavengers, e.g., hydroxocobalamin [26]. The plants are summarized in Table 1, the vasodilation effects of which are mediated via the NO–cGMP pathway. As examples, *Cynara scolymus* L. [27], *Panax ginseng* C. A. Meyer [28], and *Theobroma cacao* L. [29] can be mentioned.

##### Prostacyclin–Cyclic Adenosine Monophosphate Activation Pathway

**Figure 6:**
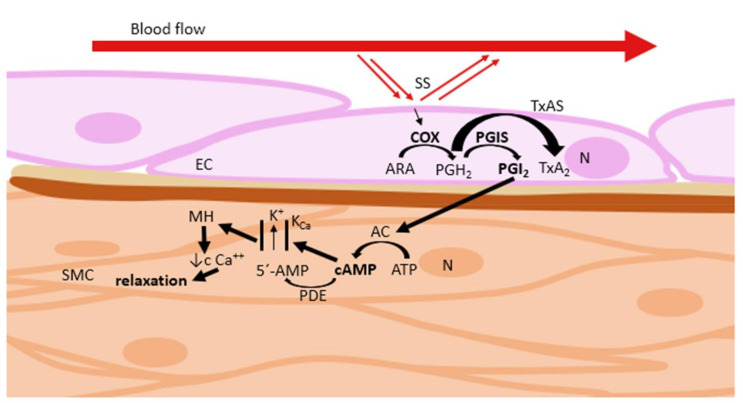
Regulation of vascular tone via prostacyclin–cyclic adenosine monophosphate pathway. AC: adenylyl cyclase; ARA: arachidonic acid; ATP: adenosine triphosphate; cAMP: cyclic adenosine monophosphate; COX: cyclooxygenase; EC: endothelial cell; K_Ca_: Ca^2+^-activated K^+^ channels; MH: membrane hyperpolarization; N: nucleus; PDE: phosphodiesterase; PGH_2_: prostaglandin H_2_; PGI_2_: prostacyclin; PGIS: prostacyclin synthase; SMC: smooth muscle cell; SS: shear stress; TxAS: thromboxane synthase.

Prostacyclin is an endogenous eicosanoid that relaxes vascular smooth muscle by stimulating the G-protein-coupled receptor. It is a vasodilator and platelet aggregation inhibitor, which activates adenylyl cyclase (AC), thereby increasing cyclic adenosine monophosphate (cAMP) levels. It also counterbalances the vasoconstrictor effect of thromboxane A_2_ (TXA_2_). Arachidonic acid (ARA) is metabolized by cyclooxygenase (COX) to form unstable prostaglandin H_2_ (PGH_2_). PGI_2_ release is further catalyzed by PGIS (Figure 6) [30,31,32]. Production of PGI_2_ is activated by endogenous substances, such as histamine, serotonin, bradykinin, and acetylcholine [32,33]. PGIS is activated by thrombin, cytokines, growth factors, and shear stress [31]. On the contrary, increased concentration of ROS inhibits PGIS activity, resulting in decreased PGI_2_ synthesis [30,31,32].

Numerous natural substances have been studied for their vasodilation effects mediated via the PGI2–cAMP pathway. Both AC inhibitor SQ22536 and protein kinase A inhibitor KT5720 can be employed to study this pathway. Another possibility is the use of analogues and antagonists of cyclic nucleotides or COX inhibitor indomethacin [26,32,33]. The plants’ vasodilation effects, which are mediated via the PGI2–cAMP pathway, are summarized in Table 2. A frequently mentioned representative of this group is *Piper truncatum* Vell [34,35].

##### Inhibition of Phosphodiesterase

**Figure 7:**
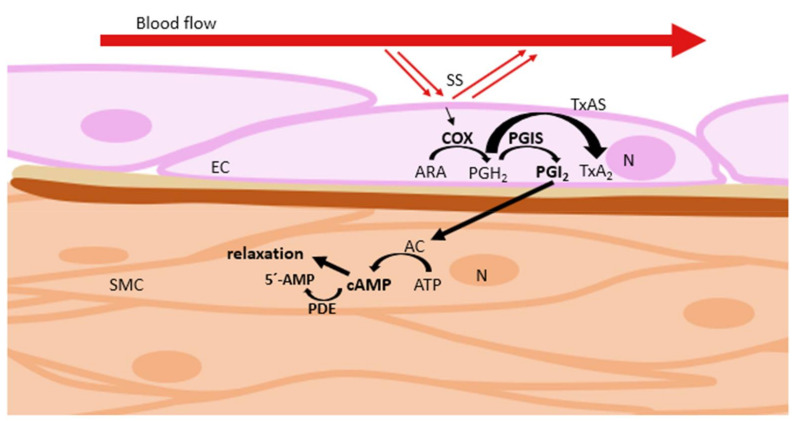
Regulation of vascular tone via inhibition of phosphodiesterase. AC: adenylyl cyclase; ARA: arachidonic acid; ATP: adenosine triphosphate; cAMP: cyclic adenosine monophosphate; COX: cyclooxygenase; EC: endothelial cell; N: nucleus; PDE: phosphodiesterase; PGH_2_: prostaglandin H_2_; PGI_2_: prostacyclin; PGIS: prostacyclin synthase; SMC: smooth muscle cell; SS: shear stress.

Cyclic nucleotide phosphodiesterases (PDEs) are enzymes regulating cellular cAMP and cGMP levels by regulation of their degradation rate. Inhibition of the PDE enzyme leads to an increase of cyclic nucleotide levels and induces vasodilation (Figure 7). The change in PDE activity, as measured by radioenzymatic assays, can elucidate the role of PDEs in the vasodilation effects of compounds in this pathway [33]. The plant metabolites that cause vasodilation via inhibition of PDE are summarized in Table 3. A model representative of such plants is *Epimedium* L. [36,37].

##### Activation of K^+^ Channels or Inhibition of Intracellular Ca^2+^ Levels

Vascular smooth muscle cell (VSMC) relaxation can be directly regulated by specific ionic channels. An important role is played by K^+^ channels. In VSMC, four different types of K^+^ channels were characterized: voltage-dependent, Ca^2+^-activated, ATP-dependent, and inward rectifier [33,38].

**Figure 8:**
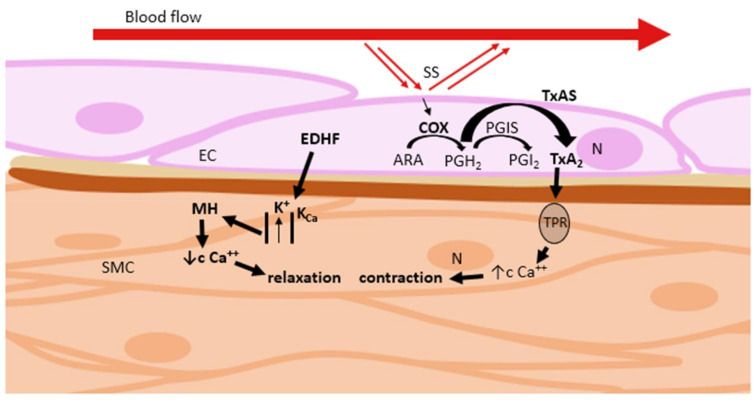
Regulation of vascular tone via activation of K^+^ channels or inhibition of intracellular Ca^2+^ levels. ARA: arachidonic acid; ATP: adenosine triphosphate; cAMP: cyclic adenosine monophosphate; COX: cyclooxygenase; EC: endothelial cell; K_Ca_: Ca^2+^-activated K^+^ channels; MH: membrane hyperpolarization; N: nucleus; PGH_2_: prostaglandin H_2_; PGI_2_: prostacyclin; PGIS: prostacyclin synthase; SMC: smooth muscle cell; SS: shear stress; TPR: thromboxane A_2_–prostanoid receptor; TxAS: thromboxane synthase.

K^+^ channels control the membrane potential in VSMC, thereby determining the activity of voltage-dependent Ca^2+^ channels (VDCC). A K^+^ channel opening leads to membrane hyperpolarization (Figure 8), resulting in closing of VDCC and preventing Ca^2+^ influx. The concentration of cytosolic Ca^2+^ is reduced, which leads to VSMC relaxation and consequent vasodilation [39]. A significant number of natural vasodilators at least partially utilize the mechanism of Ca^2+^-activated K^+^ channel activation [33,38].

Decreasing of the intracellular Ca^2+^ concentration is another possibility to induce vasodilation. Ca^2+^ enters cells through a receptor-operated Ca^2+^ channel (ROCC) or VDCC. Obstructing these channels or inhibition of Ca^2+^ release from intracellular stores lead to vasodilation [33].

Endothelium-derived hyperpolarization (EDH) represents a vasodilation system that is particularly important in small arteries, which are mostly dependent on Ca^2+^ influx during contraction. EDH is used to describe the endothelium-dependent relaxation that is non-NO and non-prostanoid in nature. This results in VSMC hyperpolarization via opening of K^+^-channels or activation of Na^+^–K^+^-ATPase [38,40].

Since 1988, several candidates have been identified as the driver of EDH, including H_2_O_2_ [7], H2S [20,41,42], epoxyeicosatrienoic acids, metabolites of ARA, K^+^ ions, electrical communication through gap junctions, and P450 epoxygenase pathway. Nowadays, E-D H_2_O_2_ is one of the major EDH in human vessels. It is generated by the dismutation of superoxide anions derived from various sources in the endothelium, including NADPH oxidase and eNOS [7]. Despite the fact that EDH evokes hyperpolarization and subsequent vasodilation (especially of small resistance vessels), higher concentrations of E-D H_2_O_2_ induce vasoconstriction by releasing COX-derived TXA_2_ [7,43].

As mentioned above, although a lot of attention is paid to NO-targeted therapy and ROS elimination (including iACEs), the evidence indicates the importance of maintaining the delicate balance between NO and EDH. Moreover, despite the fact that ROS have been considered primarily harmful for cells and tissues, physiological levels of ROS can serve as crucial signaling molecules [7].

The vasodilation is caused by either K^+^ channel activation or based on decreasing intracellular Ca^2+^ levels, which can be studied by using selective activators or blockers of specific ionic channels. Voltage–clamp or patch–clamp techniques help to elucidate the roles of particular channels and their activation or blocking in vasodilation processes. Another possibility is to study the vasodilation or vasoconstriction effect of a particular substance on isolated vessels or isolated aortic rings. Most of the present knowledge of the roles of ionic channels in vasodilation was gained in experiments using non-selective K^+^ channel blockers chloride tetraethylammonium and BaCl_2_, ATP-dependent K^+^ channel blocker glibenclamide, and voltage-dependent K^+^ channel blocker 4-aminopyridine. Various compounds affecting either Ca^2+^ influx across the plasmatic membrane via Ca^2+^ channels (such as cobalt or verapamil) or its release or re-uptake from or to the sarcoplasmic reticulum (SR Ca^2+^ channel opener ryanodine or SR Ca^2+^–ATPase blockers cyclopiazonic acid and thapsigargin) can be used in studies focusing on the changes of cytosolic Ca^2+^ availability and its impact on vascular tone [33]. The plants and their primary or secondary metabolites that lead to vasodilation via this pathway are summarized in Table 4.

All of the abovementioned substances are vasodilatory ones. Contrary to this, ET-1 and TXA_2_ are endothelium-produced vasoconstrictors. Next to them, AT II-mediated vasoconstriction is worth mentioning [32].

#### 2.2.2. Other Endothelial Functions

In addition to the previously described functions, other endothelium functions should be mentioned, such as its role in hemostasis and coagulation. Endothelial and smooth muscle cells express a variety of proteins that act both pro- and antithrombotically (intact non-wettable endothelium is an important factor in preventing intravascular hemocoagulation). Endothelial cells also participate in the regulation of inflammation [6,44].

Another endothelium function is the transport of numerous substances dissolved in blood to the subendothelial space to meet the metabolic needs of the surrounding tissues [6].

Finally, the endothelium participates in lipid metabolism on one side, while circulating lipids (fatty acids, lipoproteins) alter endothelial function on the other side. This leads to certain endothelial changes that exacerbate inflammatory processes and may promote certain diseases, such as atherogenesis [45].

## 3. Substances Affecting Vascular Tone

### 3.1. Substances with Vasoconstriction Activity

Most research is focused on substances with vasodilatory potential, since these are of high clinical relevance. Although there are also some substances with vasoconstriction activity, research studies focus on them quite rarely. In folk medicine, some plants are used for their vasoconstriction activity, e.g., *Cissus sicyoides* L. (*Vitaceae* Juss.) [46], *Nicotiana tabacum* L. (*Solanaceae* Juss.) [47,48], *Potentilla erecta* (L.) Räusch. (*Rosaceae* L.) [49], *Paspalidium flavidum* (Retz.) A. Camus (*Poaceae* Barnhart) [50], and *Haloxylon recurvum* Bunge ex Boiss. (*Amaranthaceae* Juss.) [51,52].

#### 3.1.1. Thromboxane A_2_

Thromboxane A_2_ (as well as PGI_2_) is a metabolite of ARA. For a long time, TXA_2_ was known to be released from platelets. Nowadays, it is known to be released by a variety of cells, including the endothelial ones. It stimulates platelet activation, aggregation, and proliferation, as well as vasoconstriction [53,54]. It counterbalances the effects of PGI_2_, especially in pathological situations, such as tissue injury and inflammation [54]. ARA is metabolised by COX to form unstable PGH_2_. PGH_2_ is further converted into TXA_2_ by thromboxane synthase (TXAS) [53]. TXA_2_ binds to TXA_2_–prostanoid receptor (TPR), resulting in an influx of Ca^2+^ ions and VSMC contraction [53,54]. Production of TXA_2_ can be evoked by acetylcholine, among others. TXA_2_ level reduction and TPR antagonism may be promising therapeutic targets to prevent cardiovascular disease [53,55].

As mentioned above, the production of synergic TXA_2_ and PGI_2_ is catalyzed by COX enzymes. The two COX isoforms, cyclooxygenase 1 (COX-1) and cyclooxygenase 2 (COX-2), metabolise ARA to PGH_2_, the common substrate for TXA_2_ and PGI_2_ synthesis. TXA_2_ is the predominant COX-1-derived product, in contrast to PGI_2_, which is synthetized as a result of COX-2 activation [32,56].

#### 3.1.2. Endothelin

The common name endothelin (ET) is used for three peptides, namely endothelin-1, -2, and -3 (ET-1, ET-2, and ET-3). ET-1 is the most examined endothelin and is considered the most potent vasoconstrictive substance to date. Its expression is stimulated by shear stress, thrombin, insulin, adrenaline, AT II, cortisol, and also by hypoxia; it is inhibited by NO and natriuretic peptides. ET-1 is produced by endothelial cells, smooth muscle cells, macrophages, fibroblasts, cardiomyocytes, neurons, and endocrine pancreas cells. ET-2 is formed in the ovaries and intestinal epithelial cells. ET-3 is expressed in endothelial cells, placenta, brain neurons, melanocytes, and renal tubular epithelial cells [57,58,59,60,61].

Formation of the final, biologically active ET-1 is catalyzed by endothelin-converting enzymes 1–3 (ECE 1–3), each occurring in several isoforms. ECE-1 is the major enzyme, which catalyzes all endothelin isoform formation.

Endothelin receptors ET_A_, ET_B1_, ET_B2_, and ET_C_ are G-protein-coupled receptors, differing in their affinity for individual ETs. ET-1 via ET_A_ mediates vasoconstriction (ET_A_ is expressed mainly in smooth muscle cells). Moreover, bronchoconstriction and secretion of aldosterone are mediated via ET_A_. ET_B1_ and ET_B2_ occur in both endothelial and smooth muscle cells. ET_B1_ agonist causes vasodilation by stimulating NO, PGI_2_, and EDH. On the contrary, ET_B2_ mediates vasoconstriction [57,58,59,60,61].

#### 3.1.3. Platelet-Activating Factor

Platelet-activating factor (PAF) is a phospholipid mediator, synthesis and degradation of which are catalyzed enzymatically. PAF plays a role in numerous pathophysiological reactions—it potentiates aggregation and chemotaxis, as well as formation of neutrophils, eosinophils, and monocytes. In other words, by increasing vascular permeability, it induces local inflammatory processes and edema [62].

## 4. Exogenous Substances with Vasodilation Activity

Endogenous substances with vasodilatory potential were overviewed in previous chapters. This chapter is focused on plants with a potential vasodilating effect. Table 1 to Table 4 summarize plants and their primary or secondary metabolites, in which certain effects dominate a particular signaling pathway—in Table 1 it is the NO–cGMP activation pathway, in Table 2 it is the PGI2–cAMP activation pathway, in Table 3 it is inhibition of PDE, and in Table 4 it is activation of K^+^ channels or inhibition of intracellular Ca^2+^ levels.

Numerous plants exhibiting vasodilatory effects are reported to use more than one signaling pathway. In Table 5, plant metabolites with combined mechanisms and without a dominant mechanism are summarized. Table 6 presents the plant metabolites, the effects of which have not yet been fully elucidated. Most metabolites with vasodilatory activity belong to alkaloids, flavonoids, or terpenes; additionally, stilbenes, lignans, xanthones, and coumarins are reported to have vasoactive effects. Numerous studies suggest that the most common mechanisms are interactions with the NO–cGMP pathway [33].

## 5. Conclusions

The clinical relevance of endothelial dysfunction in patients with (not only) cardiovascular disorders remains subject to investigation. Although a number of vascular and non-vascular markers of endothelial dysfunction have been proposed, inexpensive, clinically accessible, optimal, and reproducible indicators still have not been found [247]. Nevertheless, it should always be considered that numerous plants and their metabolites may impact on the endothelium and affect its physiological functions. This may become even more important if the endothelium is disordered, as can be observed in numerous diseases. Therefore, patients should be actively informed about possible interactions between the prescribed medication and various dietary supplements or folk medicines containing substances with the potential to affect endothelial functions.

Further basic science and clinical studies are needed to better inform us about the therapeutic potential of and drug interferences from plant metabolites.

## Figures and Tables

**Table 1:** Nitric oxide–cyclic guanosine monophosphate activation pathway.

Plant(s)	Primary/Secondary Metabolite(s)	Plant Part(s) Used	Citation(s)
*Allium sativum* L. (*Amaryllidaceaee* Jaume St.-Hil.)	alliin, allicin	aged garlic extract	[63,64,65,66]
*Alpinia purpurata* (Vieill.) K. Schum. (*Zingiberaceae* Lindl.)	rutin, quercetin, kaempferol-3-O-β-D-glucuronide	leaves	[67]
*Alpinia zerumbet* (Pers.) Burtt & R.M. Sm. (*Zingiberaceae* Lindl.)	catechin, epicatechin, rutin, quercetin, kaempferol 3-O-rutinoside, kaempferol 3-O-glucuronide, dihydro-5,6-dehydrokawain, 5,6-dehydrokawain	leaves	[67,68]
*Arbutus unedo* L. (Ericaceae)	tannins, afzeline, juglamine, avicularine, quercitroside, hyperoside	leaves, roots	[69]
*Caesalpinia sappan* L. (Fabaceae Lindl.)	brazilin and hematoxylin	heartwood	[70,71]
*Calicotome villosa* (Poir.) Link. (*Fabaceae* Lindl.)	chrysin	flowers, leaves	[72,73,74]
*Canavalia* DC. (*Fabaceae* Lindl.)	lectins	seeds	[75,76]
*Casimiroa* Llave & Lex (*Casimirova edulis* Llave & Lex and *Casimiroa pubescens* Ramírez) (*Rutaceae* Juss.)	hernianin, imperatorin, geranyloxypsoralen 5,6,2′,3′,4′-pentamethoxyflavon	seeds, leaves	[77,78]
*Centaurium cachanlahuen* (Mol.) Robinson (*Gentianaceae* Juss.)	xanthones	stems, flowers, leaves	[79]
*Cistus ladaniferus* L. (*Cistaceae* Juss.)	quercetin, kaempferol, myricetin	leaves	[80]
*Coptosapelta flavescens* Korth (*Rubiaceae* Juss.)	saponin, polyphenols	stems	[81]
*Crithmum maritimum* L. (*Apiaceae* Lindl.)	limonene, terpinen-4-ol, carvacrol, thymol, chlorogenic acid	flowers, stems, leaves	[82]
*Croton schiedeanus* Schlecht (*Euphorbiaceae* Juss.)	quercetin 3,7-dimethyl ether, diterpenoid and fenylbutanoid compounds	aerial parts	[83,84,85]
*Cynara scolymus* L. (*Asteraceae* Martinov)	cymaroside, luteolin, cynarin, chlorogenic acid	leaves	[27]
*Derris (Lonchocarpus) urucu* Killip & A. C. Smith (*Fabaceae* Lindl.)	isotirumalin	leaves	[86,87]
*Euterpe oleracea* C. Martius (*Arecaceae* Bercht & J. Presl)	cyanidin 3-*O*-arabinoside, cyanidin 3-*O*-glucoside, cyanidin 3-*O*-rutinoside, epicatechine, catechine homorientin, orientin, isovitexin, taxifolin deoxyhexose	fruits	[88]
*Geum japonicum* Thunberg (*Rosaceae* L.)	penta-*O*-galloyl-β-glucoside, casuariin, and 5-desgalloylstachyurin	whole plants	[89]
*Ginkgo biloba* L. (*Ginkgoaceae*)	bilobalide	leaves	[90,91]
*Inula viscosa* L. (*Asteraceae* Martinov)	cynarin, chlorogenic acid	leaves	[92]
*Magnolia grandiflora* L. (*Magnoliaceae* Juss.)	vulgarenol	flower petals	[93]
*Microdesmis keayana* J. Léonard (*Pandanaceae*)	keayanidin B, keayanin	roots	[94]
*Ocimum gratissimum* L. (*Lamiaceae* Lindl.)	eugenol	leaves	[95,96]
*Paeonia* sect. *Moutan* DC. (*Paeoniaceae*)	paeoniflorin, paeonidanin, methylpaeoniflorin, tetragalloylglucose, pentagalloylglucose	rootbark	[97]
*Panax ginseng* C. A. Meyer (*Araliaceae* Juss.)	ginsenoside-Rg1, ginsenoside Rb1	roots	[28,98,99]
*Prunella vulgaris* L. (*Lamiaceae* Lindl.)	cynaroside, luteolin, ursolic acid, betulinic acid, quercetin	flowering spike	[100,101]
*Raphanus sativus* L. (*Brassicaceae* Burnett)	sinapine thiocyanate, glucosinolates, brassinosteroids, flavonoids	seeds, leaves	[102,103]
*Rheum undulatum* L. (*Polygonaceae* Juss.)	piceatannol, tetrahydroxystilbene, resveratrol, anthraquinone derivates	rhizomes	[104,105,106]
*Saururus chinensis* (SC) Baill. (*Saururaceae*)	saucerneol, saucerneol D, machilin D	roots	[107]
*Selaginella tamariscina* (Beauv.) Spring. (*Selaginellaceae*)	amentoflavone	whole plants	[108,109]
*Solanum crispum* Ruiz & Pav (*Solanaceae* Juss.)	alkaloids, flavonoids, resins, saponins, tannins	stems, leaves	[110]
*Tabernaemontana dichotoma* Roxb. ex Wall. (*Apocynaceae* Juss.)	10-methoxyaffinisine, cathafoline, alstonisine	bark	[111]
*Tapirira guianensis* Aubl. (*Anacardiaceae* Lindl.)	triterpenoids, quercetin, myricetin glycoside, hyperoside, penta-O-galloyl-β-glucoside	leaves	[112]
*Theobroma cacao* L. (*Malvaceae* Juss.)	epicatechin, oligomeric procyanidins	seeds	[29,113,114,115,116]
*Vitis labrusca* L. (*Vitaceae* Juss.)	vitisin C, phenolic acids, anthocyanins, flavonoids	grape skin, stems	[117,118,119]
*Vitis vinifera* L. (*Vitaceae* Juss.)	vitisin C, phenolic acids, anthocyanins, flavonoids	grape skin, stems	[117,119,120,121,122]
*Ziziphus jujuba* (L.) Mill. (*Rhamnaceae* Juss.)	betulinic acid	seeds	[123]

**Table 2:** Prostacyclin–cyclic adenosine monophosphate activation pathway.

Plant(s)	Primary/Secondary Metabolite(s)	Plant Part(s) Used	Citation(s)
*Kaempferia galanga* L. (*Zingiberaceae* Lindl.)	ethyl cinnamate	rhizomes	[124]
*Piper truncatum* Vell. (*Piperaceae* C. A. Agardh)	eudesmin	leaves, stems	[34,35]
*Xylopia langdorffiana* A.St.-Hil. & Tul. (*Annonaceae* Juss.)	labdane-302	stems	[125,126]

**Table 3:** Inhibition of phosphodiesterase.

Plant(s)	Primary/Secondary Metabolite(s)	Plant Part(s) Used	Citation(s)
*Coffea arabica* L. (*Rubiaceae* Juss.)	caffeine, theobromine, theophylline, chlorogenic acid, quercetin, ferulic acid, kaempferol, rutin	seeds	[127,128,129]
*Epimedium* L. (*Berberidaceae* Juss.)	icariin	young stems	[36,37,130,131,132]

**Table 4:** Activation of K^+^ channels or inhibition of intracellular Ca^2+^ levels.

Plant(s)	Primary/Secondary Metabolite(s)	Plant Part(s) Used	Citation(s)
*Alchemilla vulgaris* L. (*Rosaceae* L.)	quercetin	aerial parts	[133,134]
*Ammi visnaga* (L.) Lam. (*Apiaceae* Lindl.)	visnagin	fruits	[135]
*Calea glomerata* Klatt. (*Asteraceae* Martinov)	flavonoids, terpenoids	aerial parts	[83,136]
*Cistus populifolius* L. (*Cistaceae* Juss.)	diterpenoids, luteolin	leaves	[137,138]
*Cymbopogon martini* (Roxb.) W.Watson (*Poaceae* Barnhart)	geraniol	leaves	[139]
*Garcinia kola* Heckel (*Guttiferae* Juss.)	kolaviron	seeds	[140]
*Gentiana kochiana* J.O.E. Perrier & Songeon (*Gentianaceae* Juss.)	gentiacaulein, gentiakochianin	roots	[141]
*Halenia elliptica* D. Don (*Gentianaceae* Juss.)	1-hydroxy-2,3,5-trimethoxy-xanthone (HM-1)	whole plants	[142]
*Hibiscus sabdariffa* L. (*Malvaceae* Juss.)	hibiscus acid, garcinia acid	calyces	[143]
*Iostephane heterophylla* (Cav.) Benth. (*Asteraceae* Martinov)	xanthorrhizol	whole plants	[144]
*Ligusticum jeholense* Nakai et Kitagawa (*Apiaceae* Lindl.)	linoleic acid, ferulic acid, ligustilide	roots, rhizomes	[145]
*Marrubium vulgare* L. (*Lamiaceae* Lindl.)	marrubiin, marrubenol	aerial parts	[146,147]
*Maxillaria densa* Lindl. (*Orchidaceae* Juss.)	gymnopusin, fimbriol A, erianthridin	whole plants	[148]
*Morinda citrifolia* L. (*Rubiaceae* Juss.)	alkaloid xeronine, phenolic compounds, sterols, flavonoids, tannins, coumarins, anthraquinones	roots	[149,150]
*Nauclea officinalis* (Pierre ex Pit.) Merr. & Chun (*Rubiaceae* Juss.)	naucline, angustine, nauclefine, naucletine	bark	[151,152]
*Peganum harmala* L. (*Zygophyllaceae*)	harmaline, harmine, harmalol	seeds	[153,154,155]
*Polygala caudata* Rehder & E.H.Wilson (*Polygalaceae* Juss.)	euxanthone	roots	[156,157]
*Prunus yedoensis* Matsum (*Rosaceae* L.)	prunetin	bark	[158,159]
*Sarcococca saligna* (D. Don) Muell.-Arg. (*Buxaceae* Dumort.)	flavonoids	whole plants	[160]
*Trachyspermum ammi* (L.) Sprague (*Apiaceae* Lindl.)	thymol, gamma-terpinene, p-cymene	seeds	[161]
*Uncaria rhynchophylla* (Miquel) Jack (*Rubiaceae* Juss.)	rhynchophylline, isorhynchophylline, hirsutine	hooks	[162,163]

**Table 5:** Combination of mechanisms without a dominant one.

Plant(s)	Primary/Secondary Metabolite(s)	Plant Part(s) Used	Citation(s)
*Agastache Mexicana* (Kunth.) Link. & Epling (*Lamiaceae* Lindl.)	tilianin, acecatin	aerial parts	[164,165]
*Alpinia henryi* K. Schum. (*Zingiberaceae* Lindl.)	cardamonin, alpinetin	seeds	[166,167]
*Alstonia scholaris* (L.) R. Br. (*Apocynaceae* Juss.)	picrinine, schloaricine, alstonamine, rhazimanine, botulin, ursolic acid, β-sitosterol	bark, leaves	[168,169]
*Alstonia macrophylla* Wall. ex G. Don (*Apocynaceae* Juss.)	vincamedine	leaves	[170]
*Andrographis paniculata* (burm. F.) Nees (*Acanthaceae* Juss.)	14-deoxyandrographolide, 14-deoxy-11,12-dihydroandrographolide	leaves	[171,172,173,174]
*Angelica dahurica* Benthman et Hooker (*Apiaceae* Lindl.)	pyranocoumarin, biscoumarin, isoimperatorin, imperatorin, phellopterin, isodemethylfuropinarine, demethylfuropinarine, decursinol	roots, rhizomes	[175,176,177,178]
*Angelica gigas* Nakai (*Apiaceae* Lindl.)	ferulic acid	roots	[179]
*Angelica keiskei* Koidz. (*Apiaceae* Lindl.)	xanthoangelol, 4-hydroxyderricin, xanthoangelol B, xanthoangelol E, xanthoangelol F	roots	[180]
*Apium graveolens* L. var. dulce DC (*Apiaceae* Lindl.)	apigenin	leaves, roots	[181,182,183]
*Bacopa monnieri* (L.) Pennel (*Plantaginaceae* Juss.)	bacoside A, bacopaside I, luteolin, apigenin	whole plants	[184,185,186,187]
*Berberis vulgaris* L. (*Berberidaceae* Juss.)	berberine	fruits, stems bark, roots	[188,189]
*Camellia sinensis* (L.) Kunzte (*Theaceae* D. Don)	epigallocatechin-3-gallate, epicatechin, epigallocatechin, epicatechin-3-gallate	green tea (leaves)	[190,191,192,193,194]
*Chenopodium ambrosioides* L.(*Amaranthaceae* Juss.)	kaempferol, quercetin, isorhamnetin, catechins, delphinidin	leaves	[195]
*Chrysanthemum morifolium* Ramat (*Asteraceae* Martinov)	luteolin-7-*O*-β-d-glucoside, apigenin-7-*O*-β-d-glucoside, acacetin-7-*O*-β-d-glucoside	flowers	[196]
*Coptis chinensis* Franch. (*Ranunculaceae* Arnott)	berberine, coptisine	rhizomes	[197,198,199,200]
*Curcuma longa* L. (*Zingiberaceae* Lindl.)	curcumane C, curcumane D, 4,5-*seco*-cadinane sesquiterpenoid	rhizomes	[201]
*Dalbergia odorifera* T. Chen (*Fabaceae* Lindl.)	butein, isoliquiritigenin, biochanin A	roots, leaves	[202,203,204,205,206,207,208]
*Dioclea grandiflora* Mart. ex Benth (*Fabaceae* Lindl.)	dioclein, floranol	roots	[209,210,211,212,213]
*Echinodorus grandiflorus* (Cham. & Schltdl.) Micheli) (*Alismataceae* Vent.)	flavonoids, diterpenes, triterpenes	leaves	[214,215,216]
*Elsholtzia splendens* Nakai (*Lamiaceae* Lindl.)	apigenin, luteolin	aerial parts	[217,218]
*Hancornia speciosa* B. A. Gomes (*Apocynaceae* Juss.)	rutin	leaves	[219]
*Liqusticum wallichii* Franchat (*Apiaceae* Lindl.)	butylidenephthalide, ligustilide, senkyunolide A, tetramethylpyrazine	rhizomes	[220,221,222,223]
*Mentha X villosa* Hudson (*Lamiaceae* Lindl.)	rotundifolone	leaves	[224,225,226,227]
*Mitragyna ciliata* aubrev. & Pellegr.(*Rubiaceae* Juss.)	mitragynine, mitraphylline, rhynophylline, flavonoids	stem bark	[228]
*Phaeanthus crassipetalus* Becc. (*Annonaceae* Juss.)	limacine, pecrassipine A, backebergine	bark, leaves	[229]
*Picrorhiza kurroa* L. (*Plantaginaceae* Juss.)	apocynin	roots	[230,231]
*Prunus serotina* Ehrh (*Rosaceae* L.)	ursolic acid, uvaol	fruits	[232]
*Schisandra chinensis* (Turcz.) Baill. (*Schisandraceae* Bl.)	schizandrin, γ-schizandrin, gomisin A	fruits (seeds)	[233,234,235]
*Scutellaria baicalensis* Georgi (*Lamiaceae* Lindl.)	baicalin	roots	[236,237]
*Senecio nutans* Sch. Bip. (*Asteraceae* Martinov)	4-hydroxy-3-(3-methyl-2-butenyl)acetophenone, 5-acetyl-6-hydroxy-2-isopropenyl-2,3-dihydrobenzofurane	aerial parts	[238]
*Thymus linearis* Benth. (*Lamiaceae* Lindl.)	thymol, carvacrol	aerial parts	[239]

**Table 6:** Not fully elucidated/not specified.

Plant	Primary/Secondary Metabolite	Plant Part Used	Citation
*Calpurnia aurea* (Ait.) Benth. (*Fabaceae* Lindl.)		seeds	[240]
*Vitex negundo* L. (*Lamiaceae* Lindl.)		aerial parts	[241]
*Ficcus saussureana* DC (*Moraceae* Dumort.)		root bark	[242]
*Prunus persica* (L.) (*Rosaceae* L.)		branches	[243]
*Satureja obovata* Lag. (*Lamiaceae* Lindl.)	eriodictyol		[244,245]
*Vernonia amygdalina* Del. (*Asteraceae* Martinov)	alkaloids, flavonoids, saponins	leaves	[246]

## Data Availability

No new data were created or analyzed in this study. Data sharing is not applicable to this article.

## Abbreviations

AC	adenylyl cyclase
ACE	angiotensin-converting enzyme
ARA	arachidonic acid
AT I	angiotensin I
AT II	angiotensin II
AT_1_R	angiotensin II receptor type-1
ATP	adenosine triphosphate
BK	bradykinin
C	calmodulin
cAMP	cyclic adenosine monophosphate
cGMP	cyclic guanosine monophosphate
CNP	natriuretic peptide C
CO	carbon monoxide
COX	cyclooxygenase
COX-1	cyclooxygenase 1
COX-2	cyclooxygenase 2
CSF	colony-stimulating factor
EC	endothelial cell
ECE-1	endothelin-converting enzyme 1
ECE-2	endothelin-converting enzyme 2
ECE-3	endothelin-converting enzyme 3
ECM	extracellular matrix
EDH	endothelium-derived hyperpolarization
E-D H_2_O_2_	endothelium-derived H_2_O_2_
eNOS	endothelial nitric oxide synthase
ER	endoplasmic reticulum
ET	endothelin
ET-1	endothelin-1
ET-2	endothelin-2
ET-3	endothelin-3
ET_A_	receptor A for endothelin
ET_B1_	receptor B1 for endothelin
ET_B2_	receptor B2 for endothelin
ET_C_	receptor C for endothelin
GTP	guanosine triphosphate
H_2_S	hydrogen sulphide
iACEs	angiotensin-converting enzyme inhibitors
i-eNOS	endothelial nitric oxide synthase inhibition
i-PGIS	prostacyclin synthase inhibition
IGF	insulin-like growth factor
iNOS	inducible nitric oxide synthase
K_Ca_	Ca^2+^ activated K^+^ channels
L-arg	L-arginine
LDL-receptor	low-density lipoprotein receptor
MH	membrane hyperpolarization
MHC II	major histocompatibility complex type 2
N	nucleus
nNOS	neural nitric oxide synthase
NO	nitric oxide
NO–cGMP	nitric oxide–cyclic guanosine monophosphate
NOS	nitric oxide synthase
PAF	platelet-activating factor
PAI	plasminogen activator inhibitor
PDE	phosphodiesterase
PGH_2_	prostaglandin H_2_
PGI_2_	prostacyclin
PGI2–cAMP	prostacyclin–cyclic adenosine monophosphate
PGIS	prostacyclin synthase
R	receptor
RAAS	renin–angiotensin–aldosterone system
ROCC	receptor-operated Ca^2+^ channels
ROS	reactive oxygen species
sGC	soluble receptor with guanylate cyclase activity
SMC	smooth muscle cell
SO Ca^2+^	store-operated Ca^2+^ channels
SR	sarcoplasmic reticulum
SS	shear stress
TGF	transforming growth factor
TPR	thromboxane A_2_–prostanoid receptor
TXA_2_	thromboxane A_2_
TXAS	thromboxane synthase
VDCC	voltage-dependent Ca^2+^ channels
VEGF	vascular endothelial growth factor
vIF	various inactive fragments
vRF	various reactive fragments
VSMC	vascular smooth muscle cell
vWf	von Willebrand’s factor
